# Performance and Hallucination Analysis of Large Language Models on European Anesthesiology Examinations: Cross-Sectional Comparative Study

**DOI:** 10.2196/95859

**Published:** 2026-09-01

**Authors:** Stefan Andrei, Thibault Giet, Alexis Belouard, Mihai Stefan, Mihai Popescu, Sébastien Tanaka, Philippe Montravers, Aurélie Gouel

**Affiliations:** 1 Department of Anesthesiology and Intensive Care, CHU Bichat Claude Bernard, Assistance Publique, Hôpitaux de Paris, Paris, Île-de-France, France; 2 Group of Data Modeling, Computational Biology, and Predictive Medicine, Applied Mathematics, Institut de Biologie de l'École Normale Supérieure, Paris, Île-de-France, France; 3 Discipline of Anaesthesiology and Intensive Care, Carol Davila University of Medicine and Pharmacy, Sos Fundeni, 258, Bucharest, București, 022322, Romania, 40 742185533; 4 2nd of Department of Anaesthesiology and Intensive Care, Institutul de Urgenţă pentru Boli Cardiovasculare "Prof.Dr. C.C. Iliescu", Bucharest, București, Romania; 5 Department of Anaesthesiology and Intensive Care, Bucharest Emergency University Hospital, Bucharest, București, Romania; 6 Diabète athérothrombose Thérapies Réunion Océan Indien (DéTROI), INSERM UMR 1188, University of Reunion Island, Saint-Pierre de La Réunion, Réunion, Réunion; 7 Physiopathology and Epidemiology of Respiratory Diseases, INSERM UMR1152, Université Paris Cité, Paris, Île-de-France, France; 8 Antibodies in Therapy and Pathology, Institut Pasteur, INSERM UMR1222, Université Paris Cité, Paris, Île-de-France, France

**Keywords:** artificial intelligence, AI, large language models, medical education, anesthesiology, intensive care medicine, hallucinations, examination, multiple-choice questions

## Abstract

**Background:**

Large language models (LLMs) have shown promising performance on medical examinations across specialties. However, comparative evaluations of current-generation LLMs across multiple European anesthesiology examinations, alongside structured assessment of hallucinations vs question-related confusion, remain lacking.

**Objective:**

This study aimed to compare the performance of 4 state-of-the-art LLMs on anesthesiology and intensive medicine examination questions and assess their hallucination rates.

**Methods:**

This computational comparative study analyzed 437 multiple-choice questions (1748 queries) from 3 sources: nurse anesthetist school examinations (infirmier anesthésiste diplômé d’État [registered nurse anesthetist]; n=100, 22.9%), European Diploma in Anaesthesiology and Intensive Care (EDAIC; n=219, 50.1%), and EDAIC On-Line Assessment (n=118, 27.0%). Each question was submitted to 4 LLMs (Claude Sonnet 4.5, Gemini 2.5 Pro, GPT-5, and Grok 4) using standardized prompts via default web interface settings. Responses were evaluated through structured consensus review by 2 examiners for accuracy, hallucinations, and question-related confusion. Statistical analysis included Friedman and Wilcoxon signed-rank tests with Holm-Bonferroni correction, the Cochran *Q* test, and generalized estimating equations.

**Results:**

Average success rates ranged from 86% (SD 18%) to 94% (SD 10%) across LLMs and examination types, exceeding the EDAIC part I passing threshold, representing substantial improvement over previously reported GPT-3.5 performance. For the EDAIC, overall intermodel differences were significant (Friedman *χ*^2^_3_=13.9; *P*=.003; *W*=0.02), with Gemini outperforming GPT-5 as the only pairwise difference. Hallucination rates ranged from 11% (11/100) to 20.1% (44/219) without significant intermodel differences. All models exceeded the EDAIC passing threshold.

**Conclusions:**

Current-generation LLMs demonstrated consistently high performance across multiple European anesthesiology examinations but continue to produce clinically relevant hallucinations, supporting their role as supervised educational tools rather than autonomous learning resources. These findings underscore the need for structured integration frameworks and systematic verification when deploying LLMs as learning tools in medical education.

## Introduction

Large language models (LLMs) have gained increasing attention in health care and medical education due to their demonstrated capacity to generate clinically relevant text, answer medical questions, and support reasoning tasks. Benchmark analyses using different standardized assessments have shown that LLMs can achieve substantial accuracy, sometimes approaching or surpassing human performance [1-4]. However, empirical evidence also reveals wide variability across tasks and domains, with persistent limitations in multimodal reasoning, complex clinical scenarios, and domain-dependent weaknesses, raising concerns about the consistency, precision, and clinical applicability of these systems [5].

LLMs may support the preparation of future health care professionals for clinical environments where AI-assisted reasoning will become increasingly prevalent. These competencies may become more relevant for modern medical practice [6]. While no universally accepted definition exists, the French National Commission on Informatics and Liberty describes LLMs as statistical models of linguistic unit distribution that predict subsequent words in a sequence [7]. This text completion mechanism, trained on vast datasets including Wikipedia, internet sources, and books, enables coherent text generation but also introduces the potential for error propagation from training data [7,8].

Despite enthusiasm regarding their potential, emerging evidence indicates that LLMs exhibit systematic vulnerabilities, including hallucinations, overconfidence, and errors in complex reasoning tasks. Operating on probabilistic text completion rather than genuine reasoning or comprehension [9], LLMs generate plausible-appearing text with perfect syntax and grammar that may diverge from contextual accuracy, user instructions, or factual knowledge [7]. These errors, collectively termed “hallucinations,” are defined by Huang et al [10] as phenomena where generated content appears nonsensical or unfaithful to the source material. Furthermore, analyses of LLM performance across cognitive domains suggest that, while these models perform adequately in factual recall and substantial clinical knowledge, their accuracy declines in tasks requiring higher-order reasoning and clinical judgment [11,12].

In a discipline requiring high-level expertise and practical knowledge, evaluating whether LLMs meet sufficient standards for pedagogical use is imperative. This study aimed to assess LLM performance and their assessment potential in anesthesiology education using standard examination questions. To our knowledge, no study has simultaneously compared multiple current-generation LLMs across different European anesthesiology examinations while systematically distinguishing hallucinations from question-related confusion.

## Methods

### Study Design

This computational comparative study evaluated 4 LLMs: Claude Sonnet 4.5 (Anthropic), Gemini 2.5 Pro (Google), GPT-5 (OpenAI), and Grok 4 (xAI), accessed via their respective publicly available web interfaces in October 2025. Models were accessed using default parameters (temperature and other generation settings were not modified). Exact internal model checkpoints were not disclosed by model providers.

### Objectives and End Points

The primary objective was to comparatively evaluate LLM performance on anesthesiology examination questions. The primary end point was the percentage of correct responses across the 4 LLMs. Secondary objectives included assessment of hallucination rates and evaluation of question clarity through LLM-identified confusion. Secondary end points comprised the proportion of serious errors (hallucinations) and the proportion of questions flagged as “confusing” by the LLMs.

### Questions and Selection

Questions were selected using a multidimensional approach drawing from (1) nurse anesthetist school examinations from Assistance Publique - Hôpitaux de Paris, designated as infirmier anesthésiste diplômé d’état (IADE; in English state-registered nurse anesthetist) questions; (2) European Diploma in Anaesthesiology and Intensive Care (EDAIC) examinations; and (3) EDAIC On-Line Assessment (OLA) practice examinations. All questions were available in online public repositories from prior examinations. None of the study authors were involved in the creation or selection of IADE examination questions.

The EDAIC, offered by the European Society of Anaesthesiology and Intensive Care, comprises written and oral components. The written examination includes 60 basic science multiple-choice questions (MCQs) followed by 60 clinical MCQs covering internal and emergency medicine; general and regional anesthesia; and specialized anesthesia, including pain management, resuscitation, and intensive care [13]. The OLA follows identical formatting for examination preparation. All questions were exclusively multiple choice to facilitate objective evaluation as MCQ responses are less subject to examiner subjectivity [14]. The IADE examination is the qualifying examination for French state-registered nurse anesthetists. The selected questions consisted of MCQs covering 5 domains (physiology, pharmacology, anesthetic techniques and implementation, procedure-related considerations, and patient-specific factors) and were administered in French, whereas EDAIC and OLA questions were administered in English. Questions were analyzed in their original language without translation.

### Study Protocol

Questions were submitted identically to the 4 LLMs following a standardized protocol. All models were queried using default web interface settings. Each question comprised a stem with 4 to 6 possible responses, with correct answers ranging from none to all options. A new conversation thread was initiated for each query to prevent cross-contamination of responses. Each individual answer option was scored as correct or incorrect, yielding a percentage success score for each question (ie, the proportion of correctly classified options out of all available options per question). This item-level scoring approach was chosen to capture partial knowledge and provide a more granular assessment of model performance than binary whole-question scoring. This scoring approach mirrors the official EDAIC examination marking scheme, in which each individual option is independently scored as correct or incorrect and partial credit is awarded accordingly.

The standardized prompt template was as follows: “[Question stem verbatim] Options: A) [option A] B) [option B] C) [option C] D) [option D] [E) and F) if applicable]. Please identify all correct answers and explain your reasoning.” This format was maintained consistently across all queries to ensure comparability.

All responses were evaluated through a structured consensus process by 2 examiners (TG and AB), who jointly reviewed each response, discussed discrepancies, and reached agreement on classification. When an LLM achieved less than 100% accuracy, joint review was conducted to distinguish between question-related confusion and true hallucination [7,10].

A hallucination was operationally defined as a response containing factually incorrect, fabricated, or unsupported medical information despite apparent comprehension of the question stem. In contrast, question-related confusion was defined as an incorrect response associated with misinterpretation of ambiguous or complex wording despite otherwise medically coherent reasoning. Representative examples illustrating these classification criteria are provided in Multimedia Appendix 1.

Hallucination and confusion analysis was performed for IADE and EDAIC questions only. The OLA was included as an external validation set for the primary end point (comparative success rates). Structured dual-reviewer classification of hallucinations and confusion for the OLA set would have required evaluation of 472 additional responses (118 × 4); the IADE and EDAIC provided 319 questions (1276 evaluated responses) across 2 distinct cognitive levels, which was considered sufficient to characterize error patterns. Given the fact that the OLA is not conceptually different from the EDAIC (it is an EDAIC simulation), it was retained as an external validation dataset for performance assessment rather than for detailed error pattern characterization.

### Statistical Analysis

Statistical analyses were performed using SPSS (version 29.0; IBM Corp) and Python (version 3.11; Python Software Foundation) with the *statsmodels* package (version 0.14), with the significance threshold fixed at a *P* value below .05 for all analyses. Quantitative variables are presented as means and SDs with 95% CIs where appropriate; qualitative variables are presented as counts and percentages. Normal distribution was assessed visually using histograms and Shapiro-Wilk testing. Results were analyzed by examination group (IADE, EDAIC, and OLA). Paired tests included Friedman and Wilcoxon signed-rank tests for quantitative variables and the Cochran *Q* test for qualitative variables. Pairwise post hoc comparisons following significant Friedman tests were performed using Wilcoxon signed-rank tests with exclusion of zero differences, and effect sizes were reported as *r* = *Z*/√*n*. To control the familywise error rate across the 6 pairwise comparisons per examination group while avoiding the excessive conservativeness of the Bonferroni method with correlated comparisons, the Holm-Bonferroni sequential procedure was applied. Holm-adjusted *P* values (denoted as “adjusted *P*”) are reported for all pairwise post hoc and domain-level comparisons and were compared against the fixed .05 significance threshold; unadjusted *P* values are reported only for omnibus tests, to which no multiplicity correction applies. The Kendall *W* was reported as the effect size for the Friedman and Cochran *Q* tests. To account for the clustered structure of the binary outcomes (each question was answered by the 4 models), pairwise intermodel comparisons of hallucination and confusion rates were performed using generalized estimating equations (GEE) with a logit link, a binomial distribution, an exchangeable working correlation structure, and robust SEs, with questions specified as clusters. All 6 pairwise model contrasts were estimated for each outcome, and the resulting *P* values were Holm adjusted.

### Ethical Considerations

This computational study did not involve human participants, patient data, biological material, or identifiable personal information. This study exclusively analyzed publicly available examination questions and AI-generated responses. According to French regulations governing biomedical research (Loi n° 2012-300 du 5 mars 2012, relative aux recherches impliquant la personne humaine, “Loi Jardé,“ modified by Ordonnance n° 2016-800 du 16 juin 2016 [15]), research not involving human participants, identifiable personal data, or biological samples falls outside the scope of mandatory ethics committee review. The Commission Nationale de l’Informatique et des Libertés (CNIL) similarly does not require declaration for research that does not process personal data. Ethics committee approval was therefore not required for the present study. No patient data were collected, stored, or analyzed at any stage of the study.

## Results

### Overview

**Figure 1.**
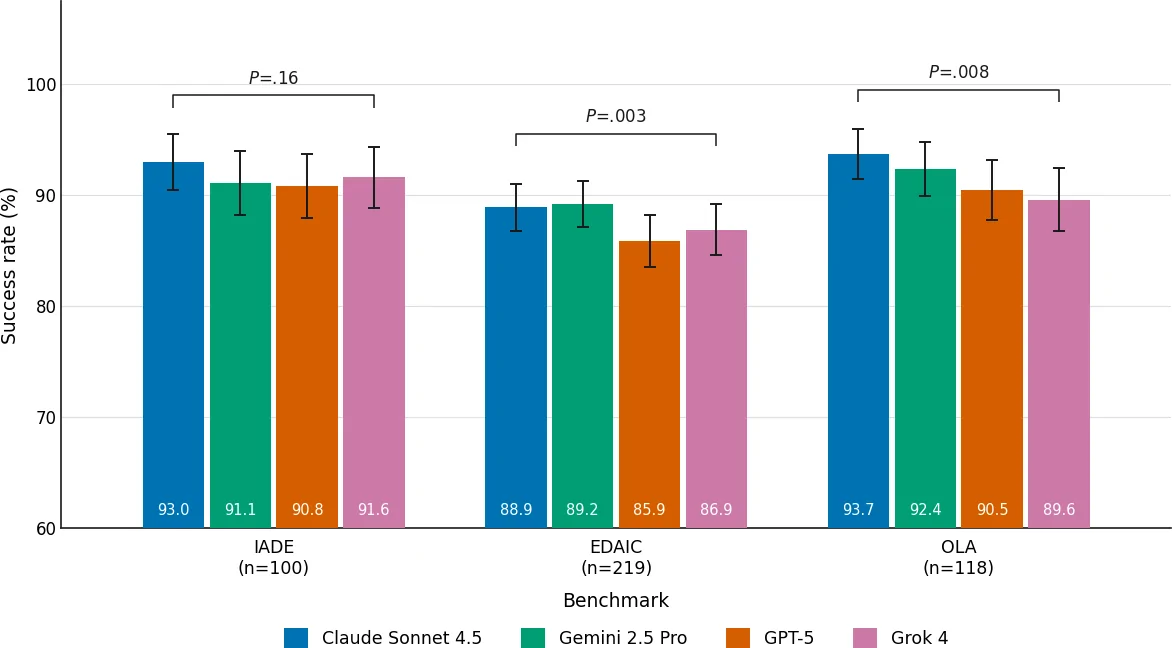
Success rates of 4 large language models across 3 anesthesiology examination types (Infirmier Anesthésiste Diplômé d'État [IADE]: n=100 questions; European Diploma in Anaesthesiology and Intensive Care [EDAIC]: n=219 questions; On-Line Assessment [OLA]: n=118 questions). Bars represent mean success rates; error bars denote 95% CIs of the mean. Brackets above each examination group indicate *P* values from the Friedman test for overall intermodel comparison.

A total of 437 questions (1748 queries) were analyzed: 100 (22.9%) IADE questions, 219 (50.1%) EDAIC questions from 2 separate examinations, and 118 (27.0%) OLA questions. Complete results are shown in Tables 1-4 and Figure 1.

**Table 1.** Success rates and hallucination and confusion rates by examination type.^a^

Examination and variables	Claude Sonnet 4.5	Gemini 2.5 Pro	GPT-5	Grok 4	*P* value
IADE^b^ (n=100 questions)					
Success rate (%), mean (SD)	93 (12)	91 (13)	91 (13)	92 (14)	.16
Hallucinations, n (%)	11 (11)	16 (16)	13 (13)	16 (16)	.34
Confusion, n (%)	8 (8)	13 (13)	14 (14)	8 (8)	.049
EDAIC^c^ (n=219 questions)					
Success rate (%), mean (SD)	89 (16)	89 (17)	86 (18)	87 (17)	.003
Hallucinations, n (%)	36 (16.4)	31 (14.2)	40 (18.3)	44 (20.1)	.10
Confusion, n (%)	51 (23.3)	53 (24.2)	67 (30.6)	59 (26.9)	.10
OLA^d^ (n=118 questions)					
Success rate (%), mean (SD)	94 (10)	93 (12)	90 (14)	90 (14)	.008

a Success rates were compared using the Friedman test, and hallucination and confusion rates were compared using the Cochran *Q* test; test statistics are reported in the Results section.

b IADE: Infirmier Anesthésiste Diplômé d’État.

c EDAIC: European Diploma in Anaesthesiology and Intensive Care.

d OLA: On-Line Assessment.

**Table 2.** Infirmier Anesthésiste Diplômé d'État examination success rates by subject domain. *P* values are Holm adjusted across the 5 domain-level Friedman tests.

Subjects	Claude Sonnet 4.5, mean (SD)	Gemini 2.5 Pro, mean (SD)	GPT-5, mean (SD)	Grok 4, mean (SD)	Adjusted *P* value
Procedure-related considerations (%; n=21)	87 (17)	90 (16)	89 (15)	86 (19)	>.99
Pharmacology (%; n=20)	92 (13)	92 (14)	90 (15)	89 (16)	>.99
Physiology (%; n=21)	99 (4)	93 (12)	93 (11)	95 (8)	.06
Techniques (%; n=14)	93 (9)	86 (12)	84 (11)	93 (9)	.16
Patient-specific factors (%; n=24)	93 (11)	93 (12)	94 (10)	95 (8)	>.99

**Table 3.** Pairwise comparisons of hallucination and confusion rates between large language models for Infirmier Anesthésiste Diplômé d'État questions: generalized estimating equation analysis.^a^

Comparisons	OR^b^ (95% CI)	Adjusted *P* value
Hallucinations		
Gemini 2.5 Pro vs Claude Sonnet 4.5	1.54 (0.84‐2.84)	.82
GPT-5 vs Claude Sonnet 4.5	1.21 (0.63‐2.30)	>.99
Grok 4 vs Claude Sonnet 4.5	1.54 (0.88‐2.70)	.78
GPT-5 vs Gemini 2.5 Pro	0.78 (0.49‐1.26)	>.99
Grok 4 vs Gemini 2.5 Pro	1.00 (0.63‐1.59)	>.99
Grok 4 vs GPT-5	1.27 (0.79‐2.05)	>.99
Confusion		
Gemini 2.5 Pro vs Claude Sonnet 4.5	1.72 (0.91‐3.24)	.34
GPT-5 vs Claude Sonnet 4.5	1.87 (0.98‐3.57)	.34
Grok 4 vs Claude Sonnet 4.5	1.00 (0.59‐1.70)	>.99
GPT-5 vs Gemini 2.5 Pro	1.09 (0.70‐1.70)	>.99
Grok 4 vs Gemini 2.5 Pro	0.58 (0.33‐1.02)	.34
Grok 4 vs GPT-5	0.53 (0.28‐1.02)	.34

a Odds ratios were estimated using generalized estimating equations with a logit link, an exchangeable working correlation structure, and robust SEs, with questions specified as clusters; *P* values are Holm adjusted across the 6 pairwise comparisons within each outcome.

b OR: odds ratio.

**Table 4.** Pairwise comparisons of hallucination and confusion rates between large language models for European Diploma in Anaesthesiology and Intensive Care questions: generalized estimating equation analysis.^a^

Comparisons	OR^b^ (95% CI)	Adjusted *P* value
Hallucinations		
Gemini 2.5 Pro vs Claude Sonnet 4.5	0.84 (0.59‐1.20)	>.99
GPT-5 vs Claude Sonnet 4.5	1.14 (0.79‐1.63)	>.99
Grok 4 vs Claude Sonnet 4.5	1.28 (0.91‐1.79)	.63
GPT-5 vs Gemini 2.5 Pro	1.36 (0.99‐1.86)	.30
Grok 4 vs Gemini 2.5 Pro	1.52 (1.08‐2.14)	.09
Grok 4 vs GPT-5	1.13 (0.80‐1.58)	>.99
Confusion		
Gemini 2.5 Pro vs Claude Sonnet 4.5	1.05 (0.76‐1.46)	>.99
GPT-5 vs Claude Sonnet 4.5	1.45 (1.03‐2.05)	.21
Grok 4 vs Claude Sonnet 4.5	1.21 (0.87‐1.70)	>.99
GPT-5 vs Gemini 2.5 Pro	1.38 (1.01‐1.88)	.21
Grok 4 vs Gemini 2.5 Pro	1.15 (0.86‐1.55)	>.99
Grok 4 vs GPT-5	0.84 (0.60-1.16)	>.99

a Odds ratios were estimated using generalized estimating equations with a logit link, an exchangeable working correlation structure, and robust SEs, with questions specified as clusters; *P* values are Holm adjusted across the 6 pairwise comparisons within each outcome.

b OR: odds ratio.

### IADE Examination Performance

IADE performance results are summarized in Tables 1 and 2. Overall performance was high across all models, with no statistically significant intermodel differences observed (Friedman *χ*^2^_3_=5.2; *P*=.16; *W*=0.02).

Questions were categorized into 5 domains: physiology (21/100, 21%), pharmacology (20/100, 20%), anesthetic techniques and implementation (14/100, 14%), procedure-related considerations (21/100, 21%), and patient-specific factors (24/100, 24%). After Holm adjustment across the 5 domain-level Friedman tests, no significant intermodel differences were observed (physiology: *χ*^2^_3_=11.1, adjusted *P*=.06, and *W*=0.18; anesthetic techniques: *χ*^2^_3_=8.3, adjusted *P*=.16, and *W*=0.20; pharmacology, procedure-related considerations, and patient-specific factors: adjusted *P*>.99). Given the small per-domain sample sizes, these analyses should be interpreted as exploratory.

### EDAIC Examination Performance

EDAIC performance results are summarized in Table 1. Overall performance remained high across all models, with significant overall intermodel differences observed (Friedman *χ*^2^_3_=13.9; *P*=.003; *W*=0.02). Claude Sonnet 4.5 and Gemini 2.5 Pro achieved the highest overall performances, whereas GPT-5 and Grok 4 showed lower mean scores.

In pairwise Wilcoxon signed-rank comparisons, Gemini 2.5 Pro significantly outperformed GPT-5 (*Z*=2.75; *r*=0.32; adjusted *P*=.04). No other pairwise comparisons were significant (adjusted *P*≥.09 in all cases).

### OLA Examination Performance

OLA performance results are summarized in Table 1. Overall performance remained high across all models, with statistically significant overall intermodel differences observed (Friedman *χ*^2^_3_=12.0; *P*=.008; *W*=0.03). Claude Sonnet 4.5 achieved the highest performance, whereas GPT-5 and Grok 4 showed lower mean scores. In pairwise Wilcoxon signed-rank comparisons, Claude Sonnet 4.5 significantly outperformed Grok 4 (*Z*=3.18; *r*=0.53; adjusted *P*=.009). No other pairwise comparisons were significant (adjusted *P*≥.08 in all cases).

### Hallucination and Confusion Analysis

Hallucination and confusion rates for each model are shown in Table 1. For IADE questions, hallucination rates did not differ significantly across models (Cochran *Q*_3_=3.38; *P*=.34; *W*=0.01). The omnibus test for confusion rates reached nominal significance (Cochran *Q*_3_=7.85; *P*=.049; *W*=0.03); however, the effect size was negligible, and no pairwise contrast was significant after Holm adjustment in the GEE analysis (Table 3; adjusted *P*≥.34 in all cases).

For EDAIC questions, neither hallucination rates (Cochran *Q*_3_=6.22; *P*=.10; *W*=0.01) nor confusion rates (Cochran *Q*_3_=6.28; *P*=.10; *W*=0.01) differed significantly across models. In the pairwise GEE analyses (Tables 3 and 4), no contrast was significant after Holm adjustment for either outcome; the largest contrast was observed for EDAIC hallucinations between Grok 4 and Gemini 2.5 Pro (odds ratio 1.52, 95% CI 1.08‐2.14; adjusted *P*=.09).

## Discussion

### Principal Findings

In this study, we compared the performance of 4 current-generation LLMs across multiple European anesthesiology examination datasets and evaluated hallucination and question-related confusion rates. All evaluated models demonstrated high overall performance, with success rates approaching or exceeding typical human passing thresholds for the EDAIC examination. Although overall intermodel differences were detected, a single pairwise difference per examination remained significant after correction, indicating that no model was consistently superior. Importantly, hallucination rates remained nonnegligible across all models, highlighting an important limitation for unsupervised educational use. Notably, LLM performance surpassed typical first-attempt pass scores for the EDAIC, which range between 60% and 75% (2025 passing scores: 67% for part A and 73.5% for part B), indicating that LLMs may outperform average examinees on standardized theoretical examinations [1,16].

The higher performance observed on IADE compared with EDAIC questions may reflect differences in cognitive complexity, as questions requiring lower-order cognitive skills (recall and comprehension) may be more amenable to current LLM capabilities than those requiring higher-order reasoning (analysis, synthesis, and evaluation) [5,17]. However, because IADE questions were administered exclusively in French whereas EDAIC and OLA questions were administered exclusively in English, the effects of examination type and language cannot be disentangled. Consequently, the observed performance differences may reflect differences in cognitive complexity, language, or examination format or a combination of these factors.

After Holm-Bonferroni correction, a single robust pairwise difference remained per examination: Gemini outperformed GPT-5 on the EDAIC, and Claude Sonnet 4.5 outperformed Grok 4 on the OLA. However, absolute performance differences between models were modest (2‐4 percentage points), suggesting that, while statistically detectable, these disparities may have limited practical significance. The clinically relevant finding is that all models performed comparably well, and hallucination rates represent a more meaningful differentiator for educational deployment.

### Comparison With Prior Work

These findings represent substantial improvement from previous reports. We evaluated GPT-3.5 in 2023 for the EDAIC part 1, finding a 70.5% success rate [1]. Other authors have documented similar performance with earlier model versions [18,19]. Our results demonstrate the rapid evolution of these technologies, consistent with other work showing progressive improvement across ChatGPT versions on medical licensing examinations worldwide [20]. In the United States, a recent exploratory study showed that ChatGPT can perform similarly to humans in standardized oral examinations as graded by American Board of Anesthesiology examiners [21], and other data show that current LLMs can surpass the minimum competency required for American Board of Anesthesiology certification [22]. Remarkable data have also emerged from various medical fields in recent years suggesting that LLMs can achieve high levels of performance in knowledge-based tasks while exhibiting important limitations in complex clinical reasoning and reliability [23,24].

Regarding hallucinations, our data do not support declaring any LLM superior. Confusion rates showed no significant intermodel differences except for a marginal effect on the IADE set (*P*=.049; *W*=0.03), which was of negligible magnitude and not corroborated by the pairwise GEE analysis. Training data may naturally contain errors that LLMs perpetuate, and users lack direct control over consulted sources, making verification challenging. Research on GPT-4o using Chilean national examination questions demonstrated that, despite strong factual recall and comprehension, the model exhibited limitations in complex reasoning and diagnostic judgment, producing unfounded medical assertions and imprecise conclusions [11]. Importantly, the hallucination rates of 11% (11/100) to 20.1% (44/219) in our study carry significant practical implications for medical education. A student relying on an LLM for self-directed learning may encounter erroneous information in approximately 1 out of every 5 to 10 queries, potentially reinforcing incorrect medical knowledge if not critically evaluated. Unlike factual errors that may be easily identified, LLM hallucinations are typically presented with high confidence and syntactic fluency, making them particularly insidious in an educational context. This underscores the necessity for supervised use and systematic cross-referencing with authoritative sources when LLMs are used as learning aids.

### Implications for Education and Practice

From an educational perspective, these findings suggest that LLMs should not be used as primary knowledge sources but rather as guided learning assistants. A pragmatic framework for safe integration may include (1) mandatory cross-verification with authoritative resources, (2) supervised use in structured teaching environments, and (3) explicit training of students to recognize and critically appraise AI-generated content. Without such safeguards, the observed hallucination rates could lead to reinforcement of incorrect medical knowledge during self-directed learning.

Our standardized prompt approach, while ensuring reproducibility, may not reflect optimal performance achievable through advanced prompting techniques. Chain-of-thought prompting, few-shot learning, or retrieval-augmented generation could improve accuracy and reduce hallucinations. Future studies should systematically evaluate the impact of prompt engineering on medical examination performance.

The high acceptance of AI tools among medical students further supports the relevance of these findings [25]. Surveys indicate widespread enthusiasm for AI integration into medical curricula, with students anticipating improvements in learning conditions and educational efficiency [26]. LLMs enable personalized educational approaches through tutorial dialogue simulation, complex concept summarization, and enhanced knowledge retention, positioning them as valuable complementary tools in medical education [20].

Beyond student support, these tools may reduce educator administrative burden by automating repetitive tasks, allowing more time for personalized student guidance and improving pedagogical quality [27,28]. The integration of LLMs into medical education raises important regulatory and policy considerations. While tools for self-directed learning may require less oversight than clinical decision support systems, educational institutions should develop clear policies regarding appropriate use, required supervision, and assessment of AI-generated content accuracy. Professional societies and accreditation bodies may need to establish guidelines for LLM use in medical education curricula.

Future studies examining open-ended questions or clinical cases would be valuable for testing LLM clinical reasoning capabilities. Given the exponential evolution of these technologies, our results will likely become obsolete within months, warranting repeated evaluation of subsequent model versions. Additionally, intervention studies evaluating the efficacy of LLM-assisted learning compared to traditional methods would help establish evidence-based recommendations for educational integration.

### Limitations

Several limitations warrant consideration. The consensus-based evaluation approach, while ensuring classification consistency between examiners, did not involve independent parallel scoring and, therefore, did not allow for formal quantification of interrater agreement through metrics such as the Cohen κ. Future studies should incorporate independent blinded evaluations to enable formal reliability assessment. Question difficulty was not formally graded. The evaluation relied on MCQs, which primarily assess knowledge recall and pattern recognition rather than complex clinical reasoning.

Questions originated from official institutions evaluating future professionals (EDAIC and IADE), and dual review reduced evaluation bias. While our substantial question sample merits expansion for enhanced statistical reliability, the exclusive use of MCQs (rather than open-ended questions) attenuated evaluation bias. Prompt formulation was standardized, with each LLM receiving identical questions in new conversation threads to prevent cross-contamination, although prior model exposure to these question banks cannot be excluded.

An important limitation is the potential presence of some examination questions or closely related content within LLM training datasets (“training data contamination”). Consequently, high performance may partially reflect memorization or prior exposure rather than genuine reasoning ability. However, several factors mitigate this concern: IADE questions were administered in French from an institutional repository with limited online dissemination, EDAIC questions are regularly updated by the examination committee, and the use of 3 distinct sources with different formats and languages reduces the likelihood of systematic contamination across all question sets. This concern has been increasingly acknowledged in the LLM benchmarking literature: prior work has demonstrated that training data contamination can artificially inflate performance on widely used medical examination benchmarks such as MedQA and US Medical Licensing Examination–style datasets, and dedicated detection methodologies have been proposed to estimate the extent of such contamination [29,30].

Additional methodological limitations include the fact that (1) questions were administered in their original language (French for the IADE and English for the EDAIC and OLA) and potential translation or language processing effects were not explored; (2) exact model version checkpoints were not documented beyond the October 2025 time frame, limiting precise reproducibility; (3) no test-retest reliability assessment was performed to evaluate response consistency—this single-query design was chosen to reflect typical real-world use patterns, where students or clinicians query an LLM once per question rather than repeatedly. However, future studies should assess response reproducibility across multiple runs.

Because the IADE questions were administered exclusively in French and the EDAIC and OLA questions were administered exclusively in English, language and examination type are inherently confounded in this design; consequently, the observed performance differences cannot be attributed to examination type alone and should be interpreted with this limitation in mind. Finally, there is a temporal limitation given the rapid LLM evolution as these results reflect October 2025 model versions.

### Conclusions

Current-generation LLMs demonstrated high performance on anesthesiology examination questions. All models substantially exceeded human passing thresholds, consistent with previously reported progressive improvements in successive LLM iterations. However, hallucination rates remained persistent across the 4 models, emphasizing the importance of user awareness regarding this phenomenon. AI-generated outputs continue to require critical verification and supervised use in educational settings, but these models represent tools with substantial pedagogical and assessment potential. These findings highlight both the educational potential and the limitations of LLMs as supplementary learning tools for medical trainees preparing for standardized examinations. Educational deployment frameworks must therefore prioritize systematic verification mechanisms over reliance on model accuracy alone.

## Supplementary material

10.2196/95859 Multimedia Appendix 1 Representative examples of hallucination and question-related confusion classifications.

## Abbreviations

EDAIC: European Diploma in Anaesthesiology and Intensive Care
GEE: generalized estimating equations
IADE: infirmier anesthésiste diplômé d’état
LLM: large language model
MCQ: multiple-choice question
OLA: On-Line Assessment
