# Supplementary material for: Performance and Hallucination Analysis of Large Language Models on European Anesthesiology Examinations: Cross-Sectional Comparative Study
Source: JMIR Form Res. 2026 Sep 1;10:e95859. doi: 10.2196/95859 (PMC13532392; doi:10.2196/95859)
Supplement: Supplementary file 1 [file formative-v10-e95859-s001.docx]

# Multimedia Appendix 1. Representative Examples of Hallucination and Question-Related Confusion Classifications

The following three examples illustrate the operational distinction between hallucination and question-related confusion as applied during the structured consensus review process described in the Methods section. Each example presents the original multiple-choice question (verbatim, in its original administration language), the large language model (LLM) response (verbatim), an English-language summary of the LLM’s key reasoning points, the reference correct answer, the answers selected by the LLM, the final classification, and the rationale supporting that classification. The three examples were deliberately selected to illustrate each of the principal classification outcomes encountered during the review process: a case in which the LLM’s medical reasoning was substantively correct despite an incorrect final answer, classified as neither hallucination nor confusion (Case 1); a case of clear hallucination, defined as factually incorrect medical content presented with affirmative tone (Case 2); and a case of question-related confusion, in which the LLM’s medical reasoning was internally coherent but the final answer was incorrect due to misinterpretation of ambiguous question wording (Case 3).

## Case 1 — Neither Hallucination nor Confusion (Correct Reasoning, Incorrect Final Answer)

**Source:** IADE examination

**LLM evaluated:** GPT-5 (OpenAI)

**Original administration language:** French

**Question (original French)**

*Parmi les propositions concernant les impératifs anesthésiques chez un patient myasthénique, laquelle ou lesquelles est (sont) vraie(s) ?*

- A – Chez le patient myasthénique, l’anesthésie locorégionale est contre-indiquée
- B – Chez le patient myasthénique, il faut éviter les médicaments dépresseurs respiratoires en prémédication
- C – Chez le patient myasthénique, il est préférable d’utiliser des halogénés
- D – Chez le patient myasthénique, le Suxaméthonium est contre-indiqué
- E – Chez le patient myasthénique, la Kétamine aggrave le bloc neuromusculaire

**Question (English translation)**

*Among the following propositions concerning anaesthetic considerations in a patient with myasthenia gravis, which is (are) true?*

- A – In the myasthenic patient, locoregional anaesthesia is contraindicated
- B – In the myasthenic patient, respiratory depressant medications should be avoided in premedication
- C – In the myasthenic patient, halogenated agents are preferable
- D – In the myasthenic patient, Suxamethonium is contraindicated
- E – In the myasthenic patient, Ketamine worsens neuromuscular blockade

**LLM response (verbatim, French)**

*Réponse directe : Les affirmations vraies sont B et, selon de nombreuses recommandations pratiques, C est acceptable mais non « préférable » absolue ; A, D et E sont fausses. En pratique : privilégier l’anesthésie locorégionale quand c’est possible (donc A est fausse), éviter les dépresseurs respiratoires en prémédication (B vraie), les halogénés peuvent être utilisés mais ne sont pas supérieurs par principe (C formulée « préférable » est discutable), le suxaméthonium n’est pas formellement contre-indiqué mais son usage est problématique et souvent évité (D fausse), la kétamine n’aggrave pas le bloc neuromusculaire et peut aider à préserver la ventilation (E fausse).*

**Key reasoning summary (English)**

GPT-5 explicitly identified that proposition B was true and provided medically accurate justifications for each option. Regarding option C, the model itself acknowledged that the use of halogenated agents is acceptable but cannot be considered "preferable" as an absolute principle, explicitly flagging this nuance. The model correctly characterised the pharmacological pitfalls of suxamethonium and the absence of evidence for ketamine worsening neuromuscular blockade. The medical reasoning was internally consistent and aligned with current anaesthesia recommendations.

**LLM-selected answers:** B and C

**Classification:** Neither hallucination nor question-related confusion

**Rationale for classification**

Although the final selection deviated from the reference answer by including option C, the LLM’s underlying medical reasoning was substantively correct: the model explicitly recognised that the wording "preferable" was debatable and that halogenated agents are merely acceptable rather than recommended. The discrepancy therefore reflects a calibration error in mapping qualified reasoning to a binary multiple-choice decision, rather than a factual fabrication (which would define hallucination) or a misinterpretation of ambiguous wording driving medically coherent but misdirected reasoning (which would define question-related confusion). The case was consequently classified as neither hallucination nor confusion, despite the incorrect final response.

## Case 2 — Hallucination

**Source:** IADE examination

**LLM evaluated:** Grok 4 (xAI)

**Original administration language:** French

**Question (original French)**

*Parmi les propositions concernant la réhabilitation rapide post-opératoire suite à une chirurgie de l’œsophage, laquelle ou lesquelles est (sont) vraie(s) ?*

- A – Une extubation précoce permet une réhabilitation rapide en postopératoire
- B – Une analgésie optimale permet une réhabilitation rapide en postopératoire
- C – Une reprise alimentaire orale à J2 permet une réhabilitation rapide en postopératoire
- D – L’ablation de la sonde nasogastrique en SSPI permet une réhabilitation rapide en post-opératoire
- E – Une kinésithérapie et mobilisation précoce à J1 permet une réhabilitation rapide en postopératoire

**Question (English translation)**

*Among the following propositions concerning enhanced recovery after oesophageal surgery, which is (are) true?*

- A – Early extubation enables enhanced postoperative recovery
- B – Optimal analgesia enables enhanced postoperative recovery
- C – Resumption of oral feeding on postoperative day 2 enables enhanced postoperative recovery
- D – Removal of the nasogastric tube in the post-anaesthesia care unit (PACU) enables enhanced postoperative recovery
- E – Early physiotherapy and mobilisation on postoperative day 1 enables enhanced postoperative recovery

**LLM response (verbatim, French)**

*Toutes les propositions A, B, C, D et E sont vraies dans le cadre des protocoles de réhabilitation rapide postopératoire (ERAS) après chirurgie de l’œsophage, car elles contribuent chacune à accélérer la récupération, à réduire les complications et à raccourcir le séjour hospitalier.*

*Reprise alimentaire orale à J2 (C) : La reprise d’une alimentation orale précoce à J2 est faisable et sûre dans les protocoles ERAS, en commençant par des liquides clairs, pour éviter la dénutrition et accélérer la récupération fonctionnelle.*

*Ablation de la sonde nasogastrique en SSPI (D) : L’ablation précoce de la sonde nasogastrique en salle de surveillance post-interventionnelle (SSPI), dès que possible après confirmation d’absence de distension ou de complications, évite les nausées et permet une reprise digestive rapide, conforme aux ERAS qui déconseillent l’usage routinier prolongé.*

**Key reasoning summary (English)**

Grok 4 selected all five propositions (A–E) as true and provided affirmative justifications for each, framing the answer in the language of established enhanced recovery after surgery (ERAS) protocols. For oral feeding (C), the model asserted that resumption on postoperative day 2 is feasible and safe within ERAS protocols. For nasogastric tube removal (D), the model asserted that removal in the post-anaesthesia care unit, as early as possible, is consistent with ERAS recommendations.

**LLM-selected answers:** A, B, C, D, E

**Classification:** Hallucination (specifically for options C and D)

**Rationale for classification**

Current enhanced recovery protocols after oesophagectomy recommend resumption of oral feeding around postoperative day 5 and removal of the nasogastric tube around postoperative day 3, not day 2 and PACU respectively as asserted by the model. The LLM’s response is therefore substantively incorrect on these two points. The affirmative and authoritative tone with which medically false content was delivered — framed within the legitimate ERAS framework, with confident pharmacological and physiological justifications — satisfies the operational definition of hallucination as factually incorrect or unsupported medical statements presented despite apparent comprehension of the question. This case represents a high-stakes hallucination because the false content would be difficult to identify as such by a learner relying on the model for self-directed preparation, given the fluent and clinically plausible framing.

## Case 3 — Question-Related Confusion

**Source:** IADE examination, Question 73

**LLM evaluated:** Grok 4 (xAI)

**Original administration language:** French

**Question (original French)**

*Parmi les propositions concernant les fonctions du tissu sanguin, laquelle ou lesquelles est (sont) vraie(s) ?*

- A – Une des fonctions du tissu sanguin est d’éliminer les éléments nutritifs
- B – Une des fonctions du tissu sanguin est de transmettre le dioxyde de carbone à tous les tissus de l’organisme
- C – Une des fonctions du tissu sanguin est d’assurer la défense de l’organisme
- D – Une des fonctions du tissu sanguin est de conduire les déchets aux organes éliminateurs
- E – Une des fonctions du tissu sanguin est de transporter les neurotransmetteurs

**Question (English translation)**

*Among the following propositions concerning the functions of blood tissue, which is (are) true?*

- A – One function of blood tissue is to eliminate nutrients
- B – One function of blood tissue is to transmit carbon dioxide to all tissues of the organism
- C – One function of blood tissue is to ensure the defence of the organism
- D – One function of blood tissue is to carry waste products to eliminating organs
- E – One function of blood tissue is to transport neurotransmitters

**LLM response (verbatim, French)**

*Les propositions vraies concernant les fonctions du tissu sanguin sont C et D.*

*La proposition E est fausse, car le transport des neurotransmetteurs n’est pas une fonction principale du tissu sanguin, qui se concentre plutôt sur les hormones et les nutriments systémiques.*

**Key reasoning summary (English)**

Grok 4 selected propositions C and D as true and provided physiologically accurate justifications: leukocytes and plasma proteins ensure immune defence (C), and blood carries metabolic waste products such as urea and creatinine to eliminating organs such as the kidneys and liver (D). The model rejected proposition E on the explicit grounds that transport of neurotransmitters "is not a principal function" of blood tissue, focused rather on hormones and systemic nutrients.

**LLM-selected answers:** C and D

**Classification:** Question-related confusion

**Rationale for classification**

This case satisfies the operational definition of question-related confusion: an incorrect response associated with misinterpretation of ambiguous question wording, despite medically coherent reasoning. The question stem ("Une des fonctions du tissu sanguin est de…" / "One function of blood tissue is to…") does not specify whether the listed function must be a principal function or whether any documented function qualifies. Grok 4 adopted a restrictive interpretation of the stem, implicitly equating "function" with "principal function," and on the basis of this implicit narrowing rejected proposition E. The medical reasoning supporting this rejection is internally coherent and physiologically defensible — transport of neurotransmitters is indeed not a principal function of blood tissue compared with oxygen, carbon dioxide, nutrients, hormones, and waste products. However, the examination key validated proposition E because the stem did not require functions to be principal. The error therefore originates in the interpretation of ambiguous question wording rather than in factually incorrect medical content (which would define hallucination) or in correct reasoning miscalibrated to a binary scoring scheme (Case 1). This case illustrates the value of distinguishing confusion from hallucination operationally, as the underlying failure modes differ substantially in their implications for the educational deployment of LLMs: hallucination signals unreliable medical content, whereas confusion signals sensitivity to question construction and wording specificity.

## Concluding Note on the Operational Distinction

These three examples illustrate the practical application of the operational definitions used in this study. Hallucination (Case 2) is characterised by factually incorrect medical content delivered with affirmative tone and apparent comprehension of the question stem; it signals a failure of the model’s underlying medical knowledge or its grounding in reliable sources. Question-related confusion (Case 3) is characterised by an incorrect final answer driven by misinterpretation of ambiguous or complex question wording, despite otherwise medically coherent reasoning; it signals sensitivity of the model to the linguistic construction of the question rather than to its medical substance. A third category encountered during the review process is illustrated by Case 1: cases in which the LLM’s medical reasoning was substantively correct and explicitly qualified, but the final discrete answer diverged from the examination key for reasons attributable to the binary scoring scheme rather than to either hallucination or confusion. Such cases were classified as neither hallucination nor confusion. The structured consensus review process used in this study aimed to maintain a conservative classification of hallucination, restricted to clear-cut factually incorrect or unsupported medical content, in order to avoid inflation of hallucination rates through inclusion of cases more accurately attributable to question construction or scoring methodology.
